# Characteristics of the optic disc in young people with high myopia

**DOI:** 10.1186/s12886-022-02719-x

**Published:** 2022-12-08

**Authors:** Fen Zhang, Xinting Liu, Yanli Wang, Qian Wang, Miaoran Zheng, Feng Chang, Xinjie Mao

**Affiliations:** 1 School of Ophthalmology and Optometry, Wenzhou Medical University, 325000 Wenzhou, Zhejiang China; 2 Department of Ophthalmology, General Hospital of the Central Theater Command of the People’s Liberation Army of China, 430070 Wuhan, Hubei China

**Keywords:** High myopia, Optic disc, OCTA

## Abstract

**Purpose:**

This study aimed to investigate the characteristics of the optic disc in adolescents and young adults with high myopia by applying optical coherence tomography angiography.

**Methods:**

A total of 112 patients with high myopia (spherical equivalent refraction (SER) ≤ -6.00 D) aged 12 to 30 years old were enrolled in this cross-sectional study. Parapapillary atrophy (PPA) and ovality index from scanning laser ophthalmoscopy images and the degree of optic disc tilt from the optic nerve head (ONH) OCT B-scans were analysed using ImageJ and MATLAB software. Peripapillary retinal nerve fibre layer thickness (pRNFLT) and radial peripapillary capillary vessel density (RPC VD) around the optic disc were obtained from the images of the optic disc angiography scan.

**Results:**

In young high myopia patients, the PPA area was positively correlated with age, axial length (AL) and pRNFLT (all *p* < 0.05) and negatively correlated with SER (*r* = -0.222, *p* = 0.020). The degree of the optic disc tilt was associated with increasing AL and pRNFLT (all *p* < 0.05). The disc area was positively correlated with AL, pRNFLT, and RPC VD (all *p* < 0.05). In the multivariate regression analysis, PPA area was independently associated with the degree of optic disc tilt and disc area. The degree of optic disc tilt was affected by AL and PPA area while the change of disc area was influenced by PPA area and pRNFLT (all *p* < 0.05).

**Conclusion:**

In young patients with high myopia, PPA area, the degree of optic disc tilt and disc area increased with AL and pRNFLT, while decreased with SER. The association between these factors was slightly different in the adolescent and young adult groups. The degree of the optic disc tilt was more associated with AL and SER in the adolescent group while disc area showed more correlated with AL and SER in the young adult group.

## Background

The prevalence of myopia and high myopia is increasing across the world, especially in Asian countries [1, 2]. However, the progression of high myopia brings a series of changes in the fundus, including the formation of PPA(parapapillary atrophy) [3], optic disc tilt or torsion [4, 5], and changes in the peripapillary retinal nerve fibre layer in thickness and blood flow density [6], which may convert to pathological myopia such as glaucoma with increasing age [7]. Eventually, pathological myopia can cause irreversible visual impairment [8].

Previous studies have found that with an increase in AL(axial length) and age, and decrease in SER (spherical equivalent refraction), the area of PPA and the degree of optic disc tilt in people with myopia will gradually increase [9–12], which may ultimately increase the risk of glaucoma [13]. It is believed that this is related to the change in optic disc morphology and the increase in the anterior surface of the peripapillary sclera and Bruch’s membrane angle [14, 15]. Previous studies have focused on adults with more stable growth and development or healthy young adults in Australia [16, 17], but the characteristics of the optic disc in high myopia with adolescents (age with fast relative growth in height) and young adults need to be further examined in the Asian population.

Therefore, this study aimed to use OCTA (optical coherence tomography angiography) to analyse optic disc morphology in young patients with high myopia, which could lead to improvements in the monitoring of fundus changes in adolescents and young adults with high myopia and help to elucidate underlying mechanism of the association between myopia and glaucoma.

## Methods

### Setting and participants

Subjects were identified from the Eye Hospital of Wenzhou Medical University, including 112 patients aged from 12 to 30 years old with high myopia in this cross-sectional study. This project was approved by the Ethics Committee of the Eye Hospital of Wenzhou Medical University and was carried out according to the principles of the Declaration of Helsinki. The inclusion criteria were as follows: SER ≤ -6.00 D, best-corrected visual acuity (BCVA) ≥ 20/20, and astigmatism lower than 1.50 D. The exclusion criteria were as follows: BCVA < 20/20, intraocular pressure (IOP) > 21 mmHg, pathological myopia, glaucoma, or a history of intraocular surgery or systemic vascular disease, such as diabetes mellitus. In the current study, only the right eye of each patient was included for statistical analysis.

### Clinical examinations

All subjects underwent an exhaustive clinical ophthalmologic examination that included subjective refraction by a trained optometrist, noncontact IOP (Full Auto Tonometer TX-F, Topcon, Japan), fundus photographs (VISUCAM 200, Carl Zeiss, Germany), optical low-coherence reflectometry (Lenstar; Haag-Streit AG, Koeniz, Switzerland), and OCTA (RTVu-XR Avanti; Optovue, CA, USA). AL, central corneal thickness (CCT), lens thickness (LT), and anterior chamber depth (ACD) were measured using optical low coherence reflectometry. SER was calculated as the spherical power plus half the cylindrical power.

### OCTA data acquisition and processing

The OCTA scans were used with the Avanti spectral domain OCT system. Optic disc angiography (scan size of 4.5 × 4.5 mm^2^) OCTA scans were acquired in two consecutive B-scans at 304 raster positions; each B-scan consisted of 304 A-scans. Good scans with a signal strength index > 6/10 for the OCTA image were included for further analysis. Scanning laser ophthalmoscopy images centred on the optic disc were acquired from the optic disc angiography scan (scan size 4.5 × 4.5 mm^2^). The PPA area and OI (ovality index) were calculated from scanning laser ophthalmoscopy images using ImageJ version 1.60 software (the National Institutes of Health, Bethesda, MD, USA). The PPA area was determined as the total number of pixels in a circumferential pattern and was then converted from pixels into square millimetres using the automatically acquired optic disc area [18, 19]. The OI was quantified by the tilt ratio of the maximum-to-minimum disc diameter; a tilted optic disc was known to have an ovality index of 1.3 or more [4]. The degree of optic disc tilt, determined by Takehiro’s new method [11, 20], was measured using a sine curve method on optic nerve head (ONH) RNFL 3.4-mm circle B scans. The amplitude of the sine curve was regarded as the degree of the optic disc tilt. The PPA area, OI, and degree of the optic disc tilt were quantified in accordance with our previous reports [12]. Peripapillary retinal nerve fibre layer thickness (pRNFLT) and radial peripapillary capillary vessel density (RPC VD) were automatically measured in the optic disc angiography scan (scan size of 4.5 × 4.5 mm^2^).

### Statistical analysis

All data were analysed with SPSS software (version 26.0; SPSS, Inc, Chicago, IL, USA) and are presented as means ± standard deviations (SD). The relationships between the PPA area, OI, degree of optic disc tilt, disc area, pRNFLT, RPC VD, SER, AL, and other parameters were analysed using Pearson’s correlations, Spearman’s correlations, and multiple linear regression analysis. *P*-values < 0.05 were considered statistically significant.

## Results

### General characteristics

A total of 112 patients were examined in the study. The mean age was 19.23 ± 4.69 years. 49 subjects were male, and 63 subjects were female. The mean SER and axial length were − 8.26 ± 1.41 D and 26.86 ± 0.94 mm, respectively. The mean PPA area, degree of the optic disc tilt and OI were 2.76 ± 1.36 mm^2^, 1.24 ± 0.13, and 34.14 ± 10.63, respectively. The general and optic characteristics of the enrolled patients are presented in Table 1.

**Table 1:** Demographic and ocular characteristics of the participants

Variable	Description
No.	112
Age, y	19.23 ± 4.69
Sex (male/female) no %	49(43.8%)/63(56.2%)
SER, D	-8.26 ± 1.41
IOP, mm Hg	15.35 ± 2.72
ACD, mm	3.25 ± 0.22
CCT, um	540.09 ± 30.16
LT, mm	3.47 ± 0.20
AL, mm	26.86 ± 0.94
Number (%) with tilted optic disc	42(37.5%)
OI	1.24 ± 0.13
Degree of the optic disc tilt	34.14 ± 10.63
PPA area, mm^2^	2.76 ± 1.36
Disc area, mm^2^	2.35 ± 0.55
Cup/Disc V, Ratio	0.32 ± 0.11
Cup/Disc H, Ratio	0.35 ± 0.11
pRNFLT, um	118.71 ± 15.41
RPC VD, %	50.58 ± 3.02

*SER* spherical equivalent refraction, *IOP* intraocular pressure, *ACD* anterior chamber depth, *CCT* central corneal thickness, *LT* lens thickness, *AL* axial length, *OI* ovality Index, *PPA* parapapillary atrophy, *Cup/Disc V* Cup/Disc vertical, *Cup/Disc H* Cup/Disc horizontal, *pRNFLT* peripapillary retinal nerve fiber layer thickness, *RPC VD* radial peripapillary capillary vessel density

### Association between optic disc characteristics and other ocular parameters in young patients with high myopia

**Fig. 1:**
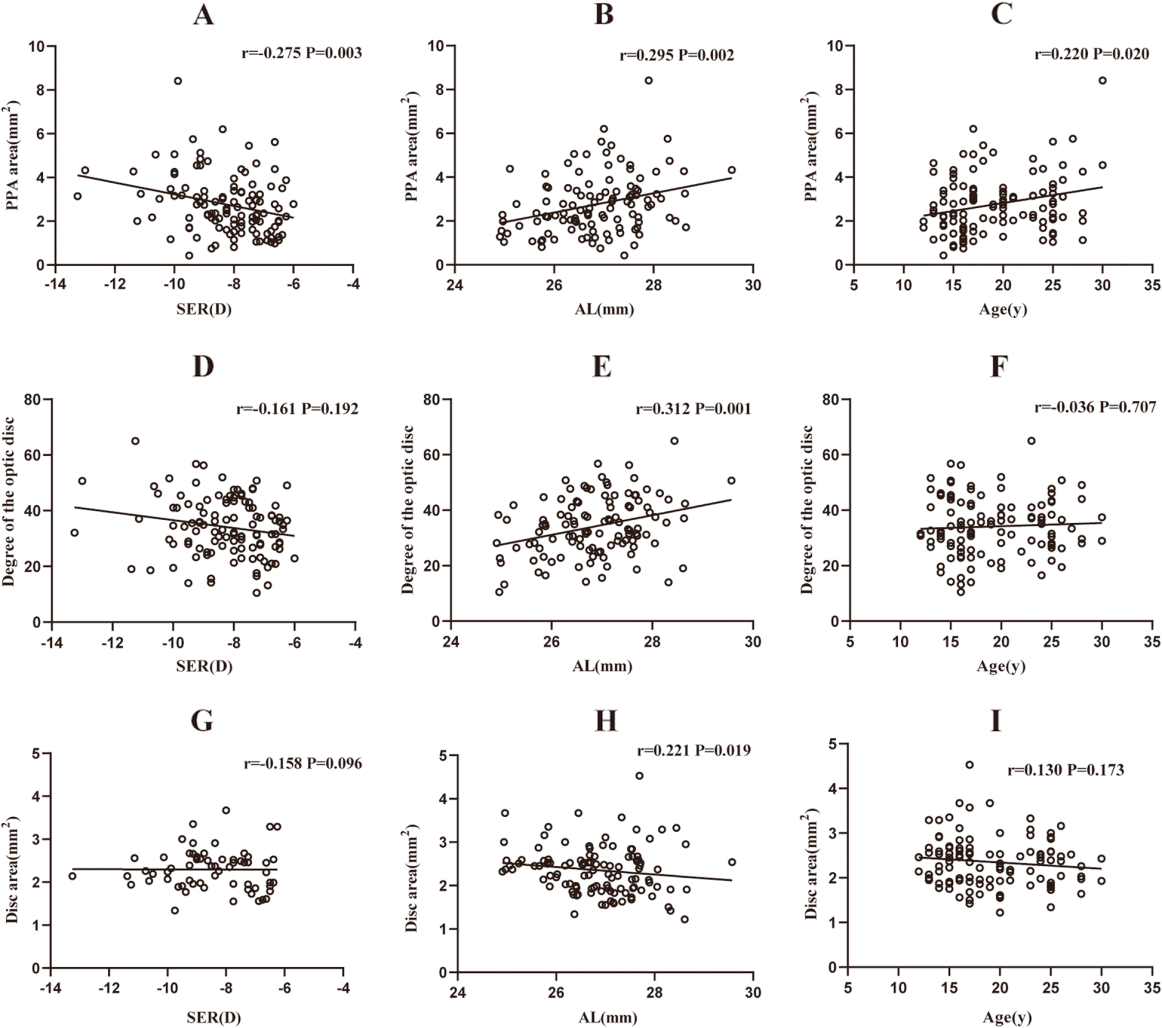
Linear correlation between the PPA area, the degree of optic disc tilt, disc area, SER, AL, and age in 112 young people with high myopia (**A**, **B**, **C**, **D**, **E**, **F**, **G**, **H**, and **I** respectively). SER Spherical equivalent refraction, AL axial length, PPA parapapillary atrophy

**Fig. 2:**
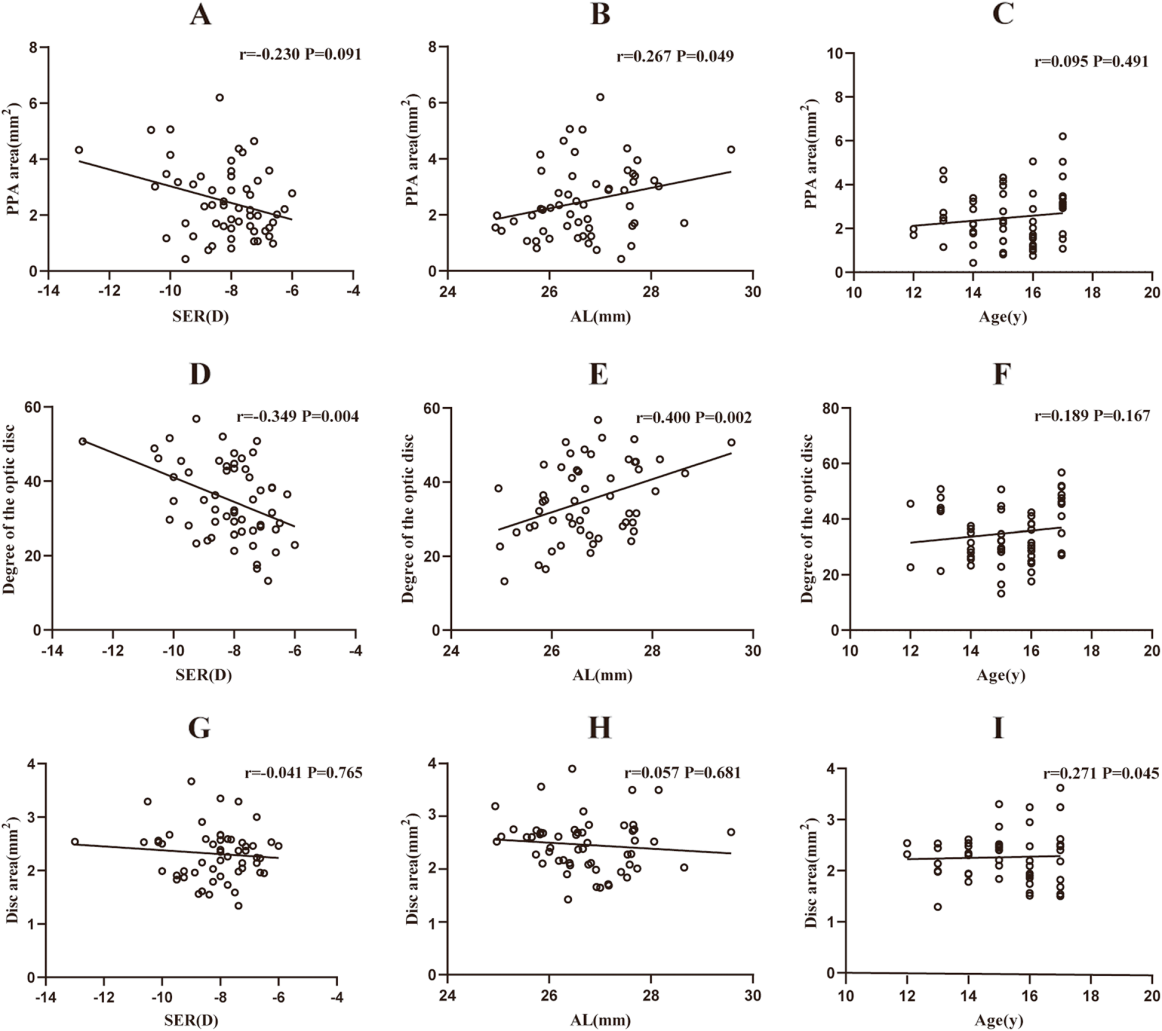
Linear correlation between PPA area, the degree of optic disc tilt, disc area and SER, AL, and age in 55 young adolescents (from 12 to 17 years old) with high myopia (**A**, **B**, **C**, **D**, **E**, **F**, **G**, **H**, and **I** respectively). SER Spherical equivalent refraction, AL axial length, PPA parapapillary atrophy

**Fig. 3:**
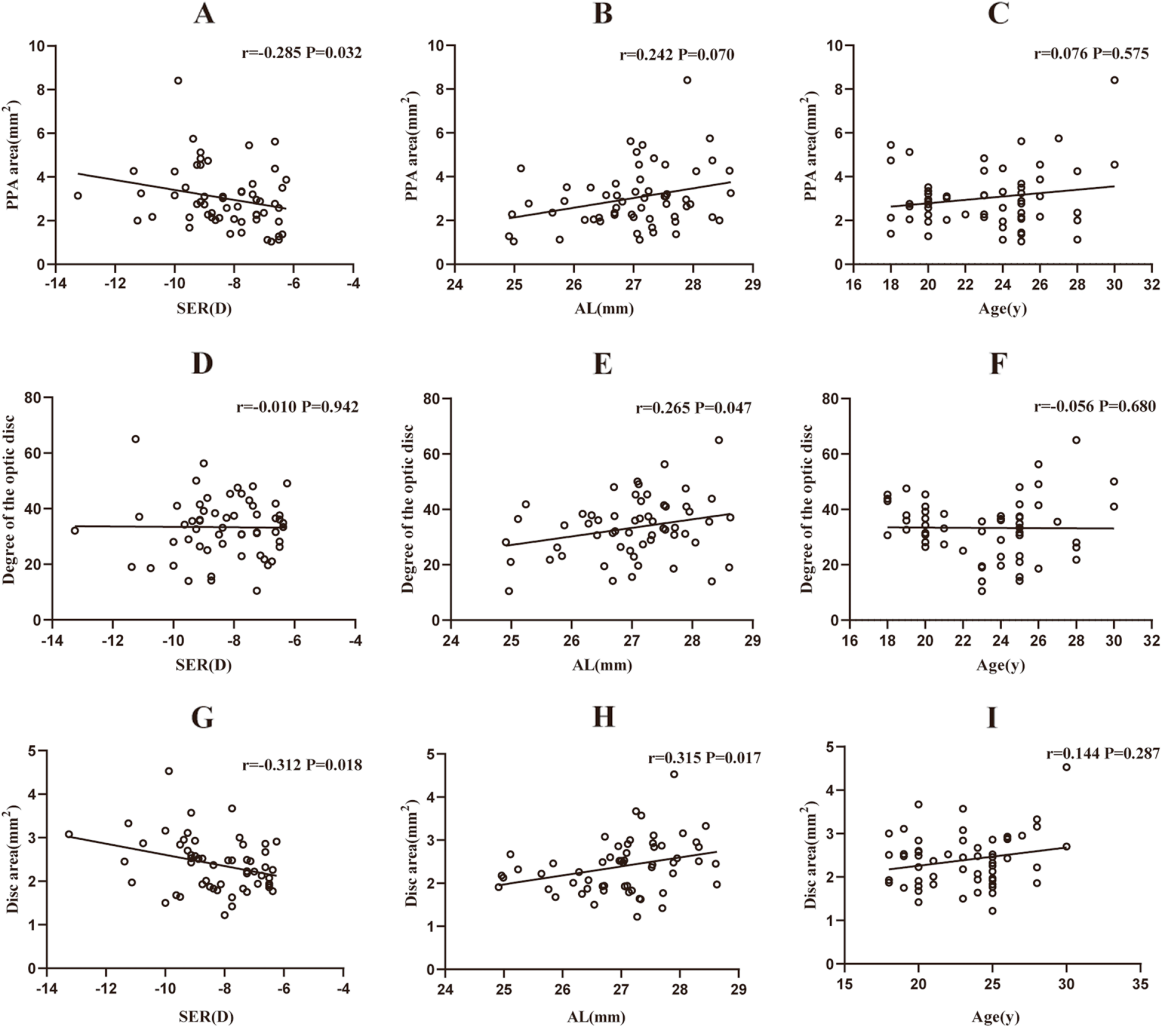
Linear correlation between the PPA area, the degree of optic disc tilt and SER, AL, and age in 57 young adults (from 18 to 30 years old) with high myopia (**A**, **B**, **C**, **D**, **E**, **F**, **G**, **H**, and **I** respectively). SER Spherical equivalent refraction, AL axial length, PPA parapapillary atrophy

Pearson’s correlation or Spearman’s correlation coefficients between the PPA area, the degree of optic disc tilt, ovality index, disc area, and other parameters in young patients with high myopia are shown in Table 2; Fig. 1. The results revealed that the PPA area was positively correlated with age (*r* = 0.222, *p* = 0.020), AL (*r* = 0.295, *p* = 0.002), the degree of optic disc tilt (*r* = 0.478, *p* < 0.001), disc area (*r* = 0.517, *p <* 0.001), and pRNFLT (*r* = 0.417, *p* < 0.001) but negatively correlated with SER (*r* = -0.275, *p* = 0.003). The degree of the optic disc tilt was positively associated with AL (*r* = 0.312, *p* = 0.001), OI (*r* = 0.214, *p* = 0.003), disc area (*r* = 0.282, *p =* 0.003) and pRNFLT (*r* = 0.278, *p* = 0.003). The OI was negatively correlated with CCT (*r* = -0.198, *p* = 0.036). The disc area was positively associated with AL (*r* = 0.221, *p* = 0.019), LT (*r* = 0.244, *p* = 0.010), pRNFLT (*r* = 0.557, *p* < 0.001) and RPC VD (*r* = 0.306, *p* = 0.001). Figure 2 shows that the PPA area and the degree of the optic disc tilt were positively correlated with AL (all *p* < 0.05), the degree of the optic disc tilt was negatively correlated with SER (*r* =-0.349, *p* = 0.004), and the disc area was positively associated with age (*r* = 0.271, *p* = 0.045) in the adolescent group. However, in the young adult group, the PPA area and disc area were negatively correlated with SER (all *p* < 0.05), and the degree of optic disc tilt and the disc area were positively associated with AL (Fig. 3).

**Table 2:** Correlation Analysis Between PPA area, Degree of the optic disc tilt, OI, Disc area, and other Ocular Parameters in young high myopia patients

	PPA area		Degree of the optic disc tilt		OI		Disc area	
	r	P	r	P	r	P	r	P
Age, y	**0.220**	**0.020**	-0.036	0.707	-0.059	0.537	0.130	0.173
Sex (male/female) no %	-0.093	0.331	0.105	0.271	-0.138	0.147	0.191	0.122
SER, D	**-0.275**	**0.003**	-0.160	0.192	0.016	0.868	-0.158	0.096
IOP, mm Hg	-0.111	0.119	-0.140	0.142	-0.117	0.218	-0.092	0.333
ACD, mm	0.049	0.607	-0.128	0.180	0.063	0.509	-0.179	0.059
CCT, um	-0.025	0.797	-0.013	0.891	**-0.198**	**0.036**	-0.064	0.505
LT, mm	0.058	0.543	-0.038	0.691	-0.092	0.336	**0.244**	**0.010**
AL, mm	**0.295**	**0.002**	**0.312**	**0.001**	-0.009	0.923	**0.221**	**0.019**
OI	0.153	0.108	**0.214**	**0.030**	-	-	0.106	0.268
Degree of the optic disc tilt	**0.478**	**<0.001**	-	-	**0.205**	**0.030**	**0.282**	**0.003**
PPA area, mm^2^	-	-	**0.478**	**<0.001**	0.153	0.108	**0.517**	**<0.001**
Disc area, mm^2^	**0.517**	**<0.001**	**0.282**	**0.003**	0.106	0.268	-	-
Cup/Disc V, Ratio	-0.130	0.173	-0.179	0.059	-0.024	0.800	0.141	0.138
Cup/Disc H, Ratio	-0.122	0.199	-0.116	0.225	0.004	0.970	-0.122	0.199
pRNFLT, um	**0.417**	**<0.001**	**0.278**	**0.003**	0.039	0.686	**0.557**	**<0.001**
RPC VD, %	**0.192**	**0.043**	-0.039	0.686	-0.061	0.525	**0.306**	**0.001**

Factors with statistical significance are shown in boldface

*SER* Spherical equivalent refraction, *IOP* intraocular pressure, *ACD* anterior chamber depth, *CCT* central corneal thickness, *LT* lens thickness, *AL* axial length, *OI* Ovality Index, *PPA* parapapillary atrophy, *Cup/Disc V* Cup/Disc vertical, *Cup/Disc H* Cup/Disc horizontal, *pRNFLT* peripapillary retinal nerve fiber layer thickness, *RPC VD* radial peripapillary capillary vessel density

### Multivariate regression analysis of PPA area, degree of optic disc tilt, and disc area in young patients with high myopia

Multivariate regression analysis was calculated to identify the independent factors associated with PPA area, the degree of optic disc tilt, and disc area in young high myopia patients, which is presented in Table 3. The results revealed that the degree of optic disc tilt and disc area were independently associated with PPA area (*p* = 0.003, *p* < 0.001, respectively). The AL and PPA area were independent predictors of the degree of optic disc tilt, while LT, PPA area, and pRNFLT were independent predictors of disc area.

**Table 3:** Multivariate Regression Analysis of PPA area, Degree of the optic disc tilt, and Disc area in young high myopia patients

	R^2^	β	P
**PPA area**	0.460		
Age		0.140	0.059
SER		-0.188	0.173
AL		0.066	0.445
Degree of the optic disc tilt		**0.240**	**0.003**
Disc area		**0.379**	**<0.001**
pRNFLT		0.146	0.117
RPC VD		0.039	0.621
**Degree of the optic disc tilt**	0.220		
AL		**0.233**	**0.010**
OI		0.152	0.080
PPA area		**0.268**	**0.019**
Disc area		-0.023	0.846
pRNFLT		0.159	0.134
**Disc area**	0.504		
LT		**0.182**	**0.009**
AL		0.082	0.268
PPA area		**0.392**	**<0.001**
Degree of the optic disc tilt		0.035	0.656
pRNFLT		**0.328**	**<0.001**
RPC VD		0.103	0.168

Factors with statistical significance are shown in boldface

*SER* Spherical equivalent refraction, *AL* axial length, *PPA* parapapillary atrophy, *OI* Ovality Index, *CCT* central corneal thickness, *pRNFLT* peripapillary retinal nerve fiber layer thickness, *RPC VD* radial peripapillary capillary vessel density

## Discussion

In recent decades, most studies have focused on the characteristics of the fundus microstructure in adults with high myopia. It has been shown that as myopia increases, axial length elongates, the thickness and blood flow of the retina and choroid decrease [21, 22], and the PPA area and the degree of optic disc tilt increase [12, 23] in many studies. However, the optic disc of the fundus of young high myopia patients remains largely unexplored in Asian populations. Therefore, this study aimed to quantitatively assess the morphological and structural characteristics of the optic disc in young people with simple high myopia using OCTA.

The current study demonstrated that the PPA area was positively correlated with AL but negatively correlated with SER. The results are consistent with the results of studies by Samarawickrama et al. [18], Mi Sun Sung et al. [24], and Liu et al. [19], all of which found that the PPA area was affected by increasing AL and SER. Simultaneously, as the age increases, the PPA area increases. This is consistent with the results of Guo [25] and his co-workers, who conducted a 5-year follow-up study of myopia and found that the PPA area in the α and β areas increased with age. Similarly, Eunoo Bak et al. [26] also demonstrated that the increase in the PPA area was significantly related to age by observing PPA changes in glaucoma and normal people over 10 years. These various studies supported that the PPA area could be correlated with the physiological age-related retinal photoreceptor and the retinal pigment epithelial cell loss rate of approximately 0.3% [26]. However, our study first showed that in the adolescent group (age from 12 to 17 years), the PPA area was only positively associated with AL and negatively associated with SER in the young adult group from 18 to 30 years old. It could be assumed that the PPA area might be caused by the stretching of the sclera and optic nerve during the progression of myopia. Therefore, the mechanisms underlying the change in PPA area among various age groups of high myopia need to be further validated.

In regard to the degree of the optic disc and OI, this study revealed that only the degree of the optic disc was positively associated with AL but not correlated with age and SER, which was not in line with several previous studies. In 2017, Li et al. [27] showed that older age, female sex, longer AL and more myopic spherical equivalent were related to a greater optic disc tilting ratio in 890 Chinese individuals aging from 7 to 70 years with bilateral high myopia. Tin A. Tun [15] showed that the angle of the anterior sclera around the optic disc increased with age by analysing the correlation between age and the morphology of the sclera around the optic disc in healthy people aged 40 to 80 years. In 2020, Wang et al. [28] demonstrated that in healthy subjects aging from 20 to 90 years with nonhigh myopia, peripapillary scleral bowing, the anterior scleral tilt rate, and Bruch’s anterior surface tilt rate increased with age, which was also inversely associated with peripapillary choroidal thickness. The difference in results among the above studies can be attributed to participants with various characteristics, such as different age ranges, refractive power, measurement instruments. And the reason why the association between optic disc morphology and AL is stronger than that with SER may be the physical changes in AL thus have a bigger impact on ocular morphology while changes in refraction are only a result of variations in ocular biometry. A larger OI was related to a stronger degree of optic disc tilt and smaller central corneal thickness but was not related to AL or SER. However, Takehiro et al. [20] reported that OI was significantly correlated with the degree of optic disc tilt. Li and his team found that a larger OI was associated with a more myopic spherical equivalent and AL [27]. The variation could be related to the different populations, highly myopic patients from 12 to 30 years old in our study, while patients from 20 to 40 years old had a wide range of spherical equivalent refraction (-14.25 ~ + 4.50 D) in Takehiro’s study and subjects aged 7 to 70 years in Li’s study. In addition, we further found that the degree of optic disc tilt was associated with a more myopic spherical equivalent in the adolescent group and with a longer axial length in the young adult group. It could be speculated that optic disc tilt may occur under the mechanism of scleral stretching by axial elongation during myopia progression among highly myopic people. It is novel that we observed the optic disc tilt were more associated with the AL and SER in the adolescent group, which might be the reason that the change in AL and SER was stronger in this age group. Further discussion as to why the association between optic disc tilt and myopia may be stronger in younger people is needed.

In our study, a larger disc area was associated with increasing AL, LT and PPA areas but not with SER. Similarly, Jost B. Jonas [29] showed that the more myopic the eye, the larger the optic disc and PPA area in high myopia. Their team further showed that a higher prevalence of optic disc enlargement was associated with a higher prevalence of circular gamma zone enlargement, higher prevalence of high myopia and increasing axial length in the Beijing Eye Study [30]. We further showed that the disc area was more associated with age in the adolescent group and with SER and AL in the young adult group. However, the underlying mechanism remains unknown, and longitudinal studies are needed to explore the characteristics of the optic disc in different age groups with high myopia.

In the structure of the retina nerve fibre layer around the optic disc, this study demonstrated that increasing PPA area and the degree of the optic disc were associated with increasing pRNFLT. In contrast, a study by Cagri Ilhan et al. [31] reported that in the high myopia group, the superior quadrant pRNFLT was significantly thinner in the tilted optic disc subgroup than in the nontilted optic disc subgroup, and the mean pRNFLT was negatively correlated with peripapillary chorioretinal atrophy extension in the inferior, superior, and nasal quadrants. Lee et al. [32] demonstrated that a thinner average pRNFLT was significantly associated with optic disc tilt, which might be more susceptible to glaucoma. However, Shin Hee Kang found that the average pRNFLT was mainly associated with AL and SER in high myopia [33]. We speculated that this might be the reason why the change in pRNFLT was likely related to the orientation of the optic nerve insertion with increasing AL. The variation in the findings among previous studies could be related to the population of highly myopic patients from aged 12 to 30 years and the measuring range for only the whole pRNFLT in our study. Our study further showed that disc area was positively correlated with pRNFLT, which was similar to the results of previous studies [34, 35]. The optic disc area significantly increased pRNFLT. Regarding the change in peripapillary vessel density, we demonstrated that only the PPA area and the disc area were correlated with the RPC VD, which is contradictory to the report of Mi Sun Sung et al. [36]. One of the possible explanations may be the different subjects and myopia groups used in different studies. In the study by Mi Sun Sung et al. [36], healthy subjects aged between 20 and 30 years and emmetropic eyes were included. In the multiple regression analyses of the optic disc characteristics, the PPA area was affected by the degree of optic disc tilt and disc area, while the degree of optic disc tilt was affected only by AL, and the disc area was influenced by pRNFLT. The underlying mechanism needs to be clarified by longer follow-up studies.

Although our study revealed several novel findings, the current study has some limitations. First, subjects with high myopia were not classified in terms of the magnitude of refractive error and the axial length. Second, there was no control emmetropic group in our study, with which can more highlight the characteristics of the optic disc in patients with high myopia. Third, this study was a cross-sectional study that simply and efficiently explored the characteristics of the optic disc, but investigating the features of the optic disc with high myopia using large longitudinal studies may provide more convincing results.

## Conclusion

In young patients with simple high myopia, the PPA area, the degree of optic disc tilt and the disc area increased with AL and decreased with SER. The association between these factors was slightly different in the adolescent and young adult groups. The degree of the optic disc tilt was more associated with AL and SER in the adolescent group while disc area showed more correlated with AL and SER in the young adult group. Consequently, monitoring the characteristics of the optic disc should be considered when managing patients with high myopia.

## Data Availability

The datasets used and analyzed during the current study are available from the corresponding author on reasonable request.

## Abbreviations

OCTA: optical coherence tomography angiography
PPA: parapapillary atrophy
OI: ovality index
pRNFLT: peripapillary retinal nerve fiber layer thickness
RPC VD: radial peripapillary capillary vessel density
SER: spherical equivalent refraction
AL: axial length
CCT: central corneal thickness
LT: lens thickness
ACD: anterior chamber depth
IOP: intraocular pressure
Cup/Disc V: Cup/Disc vertical
Cup/Disc H: Cup/Disc horizontal
ONH: optic nerve head
